# Two new methods for severity assessment of wheat stripe rust caused by *Puccinia striiformis* f. sp. *tritici*

**DOI:** 10.3389/fpls.2022.1002627

**Published:** 2022-10-03

**Authors:** Qian Jiang, Hongli Wang, Haiguang Wang

**Affiliations:** College of Plant Protection, China Agricultural University, Beijing, China

**Keywords:** wheat stripe rust, severity, disease assessment, reference range, lesion area, normal distribution method

## Abstract

Accurate severity assessment of wheat stripe rust caused by *Puccinia striiformis* f. sp. *tritici* is of great significance for phenotypic determination, prediction, and control of the disease. To achieve accurate severity assessment of the disease based on the actual percentages of lesion areas in the areas of the corresponding whole diseased leaves, two new methods were proposed for severity assessment of the disease. In the Adobe Photoshop 2022 software, the acquired images of single diseased leaves of each severity class of the disease were manually segmented, and the numbers of the leaf region pixels and lesion pixels of each diseased leaf were obtained by pixel statistics. After calculation of the actual percentages of lesion areas in the areas of the corresponding whole diseased leaves based on the obtained pixel numbers, the training sets and testing sets were constructed for each severity class by using the system sampling method with two sampling ratios of 4:1 and 3:2. Then the mean and standard deviation of the actual percentages of lesion areas contained in each training set were calculated, respectively. For each sampling ratio, two methods, one based on the midpoint value of the means of the actual percentages of lesion areas corresponding to two adjacent severity classes and the other based on the distribution range of most of the actual percentages of lesion areas, were used to determine the midpoint-of-two-adjacent-means-based actual percentage reference range and the 90%, 95%, and 99% reference ranges of the actual percentages of lesion areas for each severity class. According to the determined reference ranges, the severity of each diseased leaf in the training sets and testing sets was assessed. The results showed that high assessment accuracies (not lower than 85%) for the training sets and testing sets were achieved, demonstrating that the proposed methods could be used to conduct severity assessment of wheat stripe rust based on the actual percentages of lesion areas. This study provides a reference for accurate severity assessments of plant diseases.

## Introduction

Stripe rust (yellow rust) caused by *Puccinia striiformis* f. sp. *tritici (Pst)* is an important disease of wheat worldwide (Li and Zeng, 2002; Line, 2002; Chen, 2005; Wellings, 2011; Chen et al., 2014; Wang X. J. et al., 2014; Ali et al., 2017). It was estimated that this disease could cause yield losses of 5.47 million tons of wheat (equivalent to US$979 million) each year in the worldwide wheat-growing regions (Beddow et al., 2015). In the United States in 2000, 2001, 2002, and 2003, the total losses of wheat in the top 12 states with the most severe wheat yield losses resulting from stripe rust were approximately 1.20, 5.24, 1.06, and 11.75 million tons, respectively (Chen, 2005). As an air-borne disease, in China, wheat stripe rust has the characteristics of high epidemic frequency, wide occurrence range, and severe damage to wheat production, and it has been pandemic in wheat-growing regions for many times, especially in 1950, 1964, 1990, and 2002, reducing the yield of wheat by 6, 3.2, 1.8, and 1.3 billion kg, respectively (Li and Zeng, 2002; Wan et al., 2003; Wan et al., 2004; Wan et al., 2007). In China, wheat stripe rust is one of the most important and devastating wheat diseases and is always a serious threat to the safety of wheat production, critically affecting China’s food security (Li and Zeng, 2002; Chen et al., 2014; Wang X. J. et al., 2014; Wang et al., 2022). To carry out the surveys and monitoring of wheat stripe rust is a key way to obtain the information on the occurrences of the disease, which can provide basic supports for disease prediction, resistant variety identification, disease management, and so on.

During the surveys of wheat stripe rust, generally, the main disease indicators surveyed include incidence, severity, disease index, etc. Among these indicators, the severity is applied to describe disease intensity or the degree of infection of a plant unit (e.g., a plant, leaf, fruit, branch, stem, or other plant part) and it is of great significance for disease quantification (Nutter et al., 1991; Bock et al., 2022). For wheat stripe rust, according to the Rules for Monitoring and Forecast of the Wheat Stripe Rust (*Puccinia striiformis* West.) (National Standard of the People’s Republic China, GB/T 15795–2011), eight severity classes are classified based on the percentages of lesion areas in the areas of the corresponding whole wheat leaves. In this severity grading standard, the disease intensity between two adjacent severity classes is taken as its nearest percentage severity class, and the disease intensity of a diseased leaf with the severity lower than 1% is recorded as the severity class of 1%. Shang et al. (1990) designed a standard area diagram set for the severity assessment of wheat stripe rust, and this diagram set plays an important role in accurate severity assessment of the disease. The severity assessment of wheat stripe rust is an important part in disease surveys, concerning diseased plant phenotyping, disease prediction and forecast, and disease control decision-making. Therefore, the severity assessment should be conducted in strict accordance with the severity grading standard to ensure the assessment accuracy and to facilitate the exchange and sharing of the corresponding assessment information.

At present, the severity assessment of wheat stripe rust is conducted mainly by using visual observation method (i.e., naked eye observation method) that is heavily dependent on experienced personnel. In addition to the method above, disease severity of wheat stripe rust can be assessed by using the methods based on image processing technology (Jiang et al., 2021), remote sensing technology (Huang et al., 2004; Wang et al., 2007; Zhao et al., 2014; Wang et al., 2016), and near infrared spectroscopy technology (Li et al., 2015). In some cases, the severity of wheat stripe rust can be assessed based on the disease incidence obtained via disease survey (Dong et al., 1990).

During *Pst* infects into wheat leaves, infection sites on wheat leaves are required. The actual area occupied by each infection site may be larger than the area of each lesion with the disease symptom (usually the uredinium produced at the infection site). In the severity grading standard of wheat stripe rust (i.e., the Rules for Monitoring and Forecast of the Wheat Stripe Rust (*Puccinia striiformis* West.)) as described above, the percentage of the lesion area in the area of a whole diseased wheat leaf corresponding to one of eight severity classes is not the actual percentage of the lesion area in the area of the whole leaf. The percentage of the lesion area in the area of a whole diseased wheat leaf corresponding to a severity class in the severity grading standard is greater than the actual percentage of the lesion area in the area of the whole leaf. This makes it very difficult to accurately assess the severity of wheat stripe rust in practice. Shang et al. (1990) measured the areas of wheat leaves using a leaf area meter, and obtained the actual coverage rate of all the uredinia on a wheat leaf of each severity class using a uredinium parameters based calculating method and a method via actual measurement of the amplified image of the wheat leaf with the most severe disease symptom selected in the field. The results obtained by Shang et al. (1990) showed that the actual uredinium coverage rates for the severity classes of 1%, 5%, 10%, 20%, 40%, 60%, 80%, and 100% were 0.35%, 1.75%, 3.5%, 7%, 14%, 21%, 28%, and 35%, respectively, indicating that the actual percentage of the lesion area in the area of a whole diseased wheat leaf corresponding to one of eight severity classes is quite different from the corresponding percentage of the lesion area in the area of the whole leaf determined by using the severity grading standard of wheat stripe rust. In addition, due to the relatively small size and great shape changes of the *Pst* uredinia, it is easy to induce errors in the actual measurements of the coverage areas of the uredinia, and thus there may be some induced errors in the actual uredinium coverage rates for the severity classes obtained by Shang et al. (1990). Therefore, it is necessary to develop a more convenient and accurate method for determining the actual percentage of uredinium coverage area in a diseased wheat leaf area. Moreover, the actual uredinium coverage rate of each severity class obtained by Shang et al. (1990) is a fixed value, but most of the actual uredinium coverage rates in practice are between the fixed values of two adjacent severity classes, inducing great difficulties and inconvenience to the severity assessments. Therefore, under these circumstances, when disease severity is assessed by comparing the actual percentage of lesion area in the area of a whole diseased wheat leaf to the percentage of lesion area in the severity grading standard, great assessment errors may be induced and disease severity class may be incorrectly assessed.

The visual observation method is widely utilized to assess the severity of wheat stripe rust, it is time-consuming and laborious, and it has high requirements of experience of an assessor or a rater. When this method is utilized to carry out disease severity assessment in practice, it is not easy to conduct the assessment and to obtain accurate assessment results. Due to the influence of the human vision and experience, using this method, different assessors/raters may obtain different assessed severity class for the same diseased wheat leaf. Therefore, before carrying out the disease severity assessment in practice, an assessor or a rater is required to be trained to master the severity grading standard and the severity grading method, aiming to ensure the accuracy and reliability of the severity assessment results.

In comparison with disease severity of wheat stripe rust, it is easier to investigate disease incidence by determining whether a wheat leaf is diseased. The quantitative relationship between incidence and severity (*I-S* relationship) can be established after investigations of the incidence and severity of the disease, and then the severity can be speculated according to the incidence. Nevertheless, the *I-S* relationship is greatly affected by many factors such as the incidence, the growth stage of wheat, and the distribution of lesions on wheat leaf layers (Dong et al., 1990). Therefore, the application of the established *I-S* relationship equation/model has great limitations, limiting the application of the severity assessing method based on the disease incidence.

Studies on severity assessment of wheat stripe rust based on remote sensing technology, near infrared spectroscopy technology, image processing technology, and other information technologies, have been paid attention to. The severity assessment of wheat stripe rust based on remote sensing technology and near infrared spectroscopy technology is still in the experimental research phase. Due to the high price of the required instruments and the low practical applicability, the related methods based on remote sensing technology and near infrared spectroscopy technology are rarely applied in practical productions. With the rapid development of image acquisition technology and image processing technology, more and more recognition methods (Li et al., 2012; Wang M. L. et al., 2014; Guo et al., 2015; Hu et al., 2018) and severity assessment methods (Bao et al., 2021; Jiang et al., 2021) of wheat stripe rust based on image processing technology are utilized in research and practical applications.

At present, the methods based on image processing technology to assess the severity of wheat stripe rust can be divided into two categories; one is to directly identify the severity classes based on the extracted disease image features (Bao et al., 2021), and the other is to segment the lesion/lesions, calculate the lesion area and the area of a whole diseased wheat leaf (or the number of the lesion pixels and the number of pixels of the whole diseased leaf), calculate the actual percentage of the lesion area in the area of the whole diseased leaf, and assess the severity of the corresponding diseased leaf by comparing the actual percentage of the diseased area to the percentages for the eight severity classes in the disease severity grading standard (Jiang et al., 2021). In the current research and applications of plant disease severity assessment by using image processing technology, the situation that the percentage of the lesion area in the area of a whole diseased plant unit corresponding to each severity class in the severity grading standards of some plant diseases (such as wheat stripe rust and wheat leaf rust caused by *Puccinia triticina*) is not the actual percentage of the lesion area in the area of the whole diseased plant unit, is not taken into account. Thus the accuracies of the severity assessments of these plant diseases based on image processing technology are seriously affected, resulting in great errors or complete errors in the disease severity assessments. This is also the main reason for the low accuracy obtained in assessing the severity of these plant diseases based on the ratio of lesion area to the total area of a plant unit by using image processing technology, which limits the practical applications of the related technology.

To solve the difficulties in assessing the severity of wheat stripe rust and the problems in severity assessment of the disease based on the actual percentage of lesion area in the area of the corresponding whole diseased wheat leaf, and to improve the severity assessment accuracy, it is necessary to explore a simple, easy-to-operate, and rapid method with high accuracy for assessing the disease severity, which is of great significance for the survey, monitoring, prediction and forecast, and control of the disease. In this study, by using image processing software, the leaf region and lesion region in the acquired image of each single diseased wheat leaf were obtained via image segmentation operations, and the numbers of the lesion pixels and the pixels of the whole diseased leaf were achieved by pixel statistics. Then the actual percentage of the lesion area in the area of the whole diseased leaf was calculated, and the mean of the actual percentages of lesion areas corresponding to each severity class was calculated subsequently. Based on the midpoint value of the means of two adjacent severity classes, the reference range of the actual percentages of lesion areas corresponding to each severity class was determined for severity assessments. Furthermore, by referring to the method for determining the reference ranges in the field of medicine, the reference ranges at different levels (90%, 95%, and 99%) of the actual percentages of lesion areas corresponding to each severity class were estimated based on the distribution range of the actual percentages of lesion areas of most of the diseased wheat leaves belonging to each severity class. According to the midpoint-of-two-adjacent-means-based actual percentage reference ranges and the reference ranges at different levels for the eight severity classes, the severity of each of the acquired diseased wheat leaves with the percentages of lesion areas was assessed, and the assessment performance of each reference range was evaluated by using the assessment accuracy. In this study, it is aimed to explore two new methods for severity assessment of wheat stripe rust based on the actual percentages of the lesion areas in the areas of the corresponding whole diseased leaves, to provide a reference for severity assessments of plant diseases based on the ratios of lesion areas to the total areas of plant units, and to provide supports for the automatic severity assessments of plant diseases based on image processing technology.

## Materials and methods

**Figure 1:**
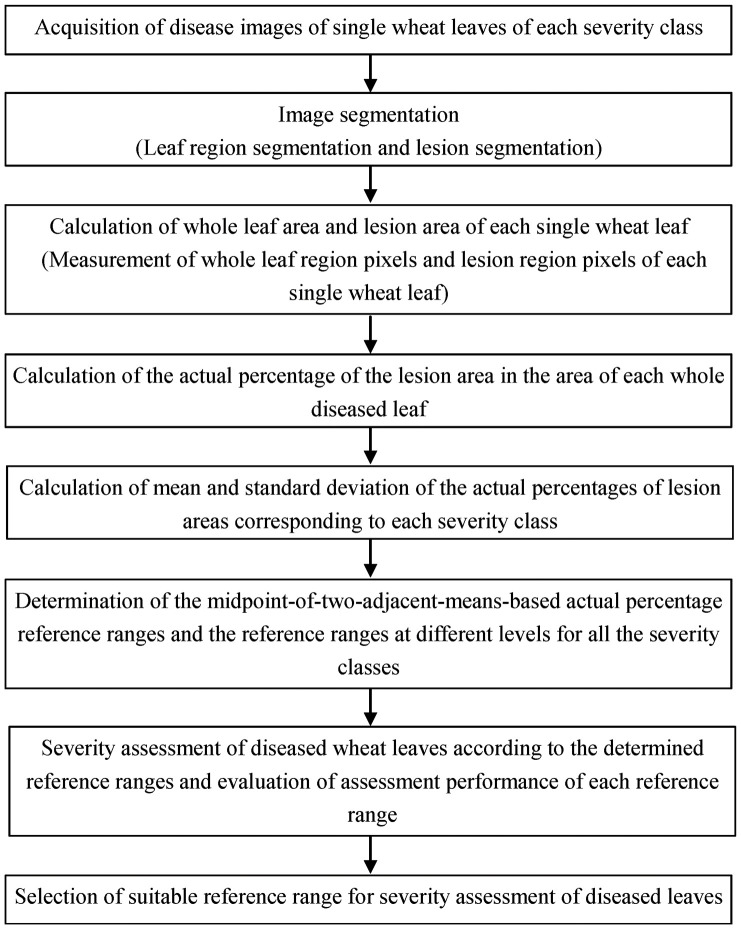
Work flow diagram for determining the reference ranges for disease severity assessment based on the actual percentages of lesion areas corresponding to each severity class and assessing the severity of wheat stripe rust.

In this study, two new methods for severity assessment of wheat stripe rust were developed according to the procedures and steps as shown in **Figure 1**, and then the constructed data sets were used to evaluate the new methods.

### Acquisition of single wheat leaf images of each severity class of wheat stripe rust

According to the Rules for Monitoring and Forecast of the Wheat Stripe Rust (*Puccinia striiformis* West.) as described above, wheat leaves with typical symptoms of wheat stripe rust with severity levels of 1%, 5%, 10%, 20%, 40%, 60%, 80%, and 100% were collected from the diseased wheat plants that were obtained by using artificial spray inoculation method in Shangzhuang Experimental Station of China Agricultural University, Beijing, China and an artificial climate chamber in the Laboratory of Macro-Phytopathology, China Agricultural University, Beijing, China. Each diseased leaf was expanded as flat as possible and fixed on a sheet of A4 white paper with the lesion side facing up by using double sided sticky tape. Images of the diseased leaves were taken with a Nikon D700 digital camera (Nikon Corp., Tokyo, Japan), a HUAWEI P30 smartphone, and an iPhone 6S smartphone, and the sizes of the corresponding acquired images were 4256×2832, 3648×2736, and 4032×3024 pixels, respectively. One image was taken for each diseased leaf, 50 single diseased leaves of each severity class were used to be photographed, and a total of 400 single diseased leaf images were acquired. All the acquired images were in the JEPG format.

### Manual image segmentation and pixel statistics of leaf regions and lesion regions of diseased wheat leaves

**Figure 2:**
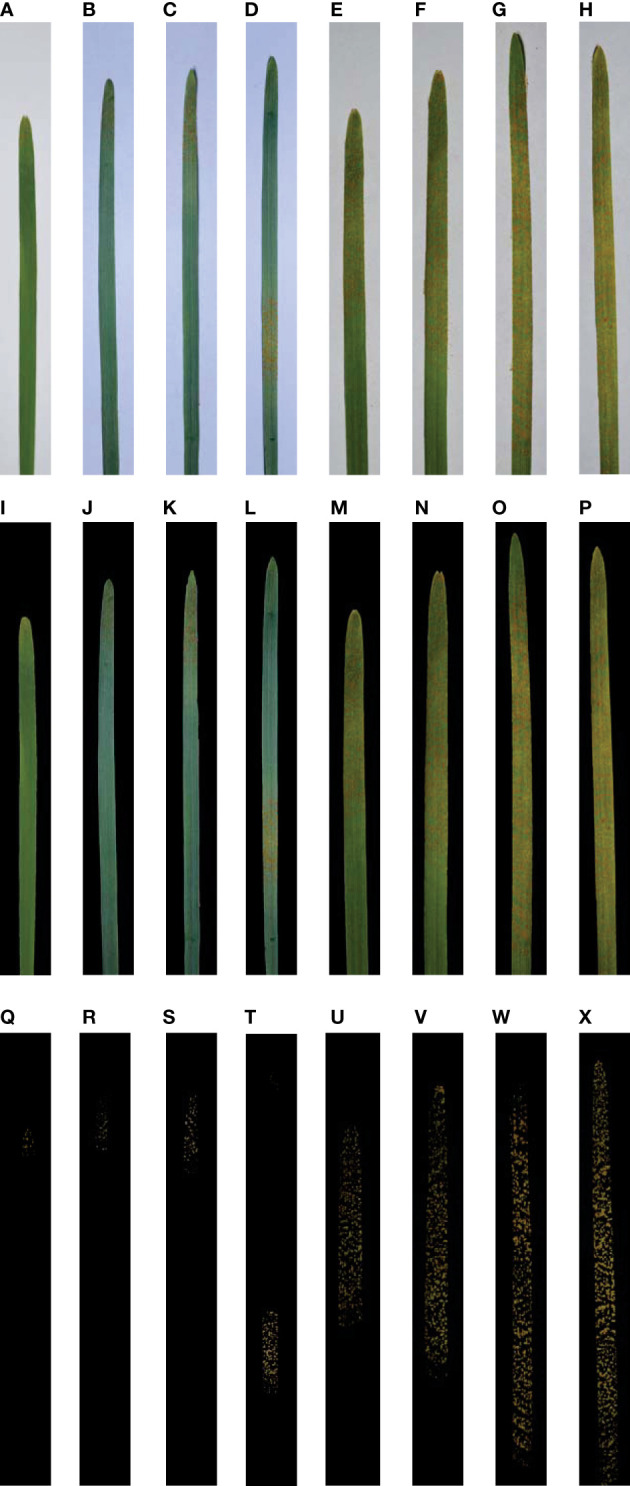
Single wheat leaf images of each severity class of wheat stripe rust and the corresponding leaf region images and lesion images after segmentation. All the images were shown after being cropped uniformly so that they could be demonstrated clearly. **(A–H)** Single diseased wheat leaf images of the severity classes of 1%, 5%, 10%, 20%, 40%, 60%, 80%, and 100%, respectively; **(I–P)** Segmented images of leaf regions for the single diseased wheat leaf images of the severity classes of 1%, 5%, 10%, 20%, 40%, 60%, 80%, and 100%, respectively; **(Q–X)** Segmented lesion images for the single diseased wheat leaf images of the severity classes of 1%, 5%, 10%, 20%, 40%, 60%, 80%, and 100%, respectively.

Manual image segmentation and pixel statistics of leaf regions and lesion regions of diseased wheat leaves were conducted by using the Adobe Photoshop 2022 software (Adobe Systems Incorporated, San Jose, CA, USA). In the software, a single leaf image (as shown in **Figures 2A–H**) of wheat stripe rust was opened, then the whole leaf region was selected with the quick selection tool, and subsequently the pixel number of the whole leaf region was viewed in the histogram panel and was recorded in a sheet in Microsoft Excel 2016. Inverse selection was carried out, then the selected region was filled with black color, and, finally, the image was saved in the JPEG format and the TIFF format, respectively (as shown in **Figures 2I–P**). When the quick selection tool was used, the ‘Enhance Edge’ was not selected, and for the brush options, in most cases, the size was set to 5 pixels, the hardness was set to 0%, the spacing was set to 25%, the angle was set to 35°, and the roundness was set to 100%. After completing the image segmentation of the diseased leaf, the diseased leaf layer was duplicated to form a new layer, and the original diseased leaf layer was named background and the new layer was named Layer 1 in the Adobe Photoshop 2022 software. Then the background layer was hidden, and Layer 1 was shown and selected. Repeatedly, the magic wand tool was used to select the non-lesion regions and the corresponding selected regions were filled with black color, so as to complete the initial segmentation of the lesion/lesions. After completing the initial segmentation, if there was still any non-lesion region that was not shown as black, the region was circled by using the lasso tool and was subsequently filled with black color, so as to complete the secondary segmentation of the lesion/lesions. The background layer was shown, Layer 1 was selected, and whether any lesion region was completely segmented or not was checked by repeatedly showing and hiding Layer 1. If there was still any lesion region shown as black, Layer 1 was selected and hidden, the background layer was shown, and then the region was circled by using the lasso tool and was subsequently removed. Until any lesion region was completely segmented, the non-lesion region was clicked by using the magic wand tool, then the inverse selection was carried out, and subsequently the pixel number of the lesion region/regions was viewed in the histogram panel and was recorded in the sheet in Microsoft Excel 2016. Finally, Layer 1 was saved in the JPEG format and the TIFF format, respectively (as shown in **Figures 2Q–X**). When the magic wand tool was used, the sample size was set to point sample and the tolerance value was set to a number between 0 and 35. According to the actual selection of the lesion region, the tolerance value can be adjusted and the ‘Contiguous’ option can be selected. When the lasso tool was used, the feather value was set to 0 pixel. In all the above processes, the options ‘Anti-alias’ and ‘Sample All Layers’ were not selected. The numbers of the lesion pixels and the whole diseased leaf pixels for each diseased wheat leaf image were obtained by using the method as described above.

### Calculation of the actual percentage of the lesion area in the area of each whole diseased wheat leaf

For each diseased wheat leaf image, based on the pixel number of the whole leaf region and the pixel number of the lesion region/regions recorded in the sheet in Microsoft Excel 2016, the percentage of the pixel number of the lesion region/regions in the pixel number of the whole leaf region, i.e., the actual percentage of the lesion area in the area of the whole diseased leaf, was calculated according to the following Formula (1).


(1) $$r=\frac{A_{\text{d}}}{A_{\text{l}}}\times 100\%$$

where *r* is the actual percentage of the lesion area in the area of the whole diseased leaf for a single diseased wheat leaf, A_d_ is the pixel number of the lesion region/regions in the single diseased wheat leaf image, and A_l_ is the pixel number of the whole leaf region in the single diseased wheat leaf image.

### Normal distribution tests on the data of the actual percentages of lesion areas corresponding to each severity class and the data of the reconstructed data sets after sampling

Normal distribution test on the data of the actual percentages of lesion areas in the corresponding whole leaf areas at the severity level of 1%, 5%, 10%, 20%, 40%, 60%, 80%, or 100% was conducted by using the UNIVARIATE procedure in the SAS 9.4 software (SAS Institute Inc. Cary, NC, USA). The results showed that 50 actual percentages of the lesion areas in the corresponding whole leaf areas for each severity class had a normal distribution. After 50 actual percentages of the lesion areas in the corresponding whole leaf areas for each severity class were sorted from large to small, the training and testing sets were constructed based on the data sampled from the 50 actual percentages by using the system sampling method with the ratio of the number of specimens in the training set to the number of specimens in the testing set equal to 4:1 or 3:2. For the sampling ratio of 4:1, the corresponding constructed training and testing sets were recorded as Train40_R_ and Test10_R_, respectively. Train40_R_ was composed of the 40 specimens obtained by using the system sampling method with the sampling ratio equal to 4:1 when the severity class was *R*, and Test10_R_ was composed of the 10 remaining specimens. For the sampling ratio of 3:2, the corresponding constructed training and testing sets were recorded as Train30_R_ and Test20_R_, respectively. Train30_R_ was composed of the 30 specimens obtained by using the system sampling method with the sampling ratio equal to 3:2 when the severity class was *R*, and Test20_R_ was composed of the 20 remaining specimens. *R* was the percentage of the lesion area in the area of the whole diseased leaf of the corresponding severity class in the severity grading standard of wheat stripe rust, so the value of *R* could be 1%, 5%, 10%, 20%, 40%, 60%, 80%, or 100%. Normal distribution tests on the data of the training sets (Train40_R_ and Train30_R_) for the severity class of *R* were conducted by using the UNIVARIATE procedure in the SAS software. The results showed that the actual percentages of lesion areas contained in each training set had a normal distribution.

### Calculation of the mean and standard deviation of the actual percentages of lesion areas corresponding to each severity class

The mean ( $\bar{r}$ ) and standard deviation (*s*) of the actual percentages of lesion areas contained in each training set (Train40_R_ or Train30_R_) for the severity class of *R* were calculated, respectively. The value of $\bar{r}$ for each severity class was treated as the representative value of the actual percentage of lesion area in the area of a whole diseased wheat leaf of the corresponding severity class.

### Determination of the reference ranges of the actual percentages of lesion areas in the corresponding whole leaf areas for all the severity classes

The reference ranges of the actual percentages of lesion areas in the corresponding whole leaf areas for all the severity classes of wheat stripe rust were determined by using the following two methods.

Method 1: The actual percentage reference range corresponding to each severity class was determined by taking the midpoint value (r_midpoint_) of the means of the actual percentages of lesion areas of two adjacent severity classes as the demarcation point, and this midpoint-of-two-adjacent-means-based actual percentage reference range was treated as one kind of the reference ranges of the actual percentages of lesion areas in the corresponding whole leaf areas for the severity class. Based on the actual percentages of lesion areas contained in each training set (Train40_R_ or Train30_R_), the midpoint value r_midpoint_ of the means of two adjacent severity classes was treated as the demarcation point, and then the r_midpoint_ value was regarded as the upper limit of the actual percentages of lesion areas corresponding to the lower severity class in the two adjacent severity classes and was regarded as the lower limit of the actual percentages of lesion areas corresponding to the higher severity class. For the lowest severity class (1%), the lowest actual percentage of lesion area in the corresponding whole diseased leaf is greater than 0%. Thus the midpoint-of-two-adjacent-means-based actual percentage reference ranges corresponding to the severity class of *R* based on the training sets Train40_R_ and Train30_R_ were determined for the severity assessment of wheat stripe rust. The actual percentage of the lesion area corresponding to demarcation point was calculated according to the following Formula (2).


(2) $$r_{\text{midpoint}}=\frac{\bar{r_{a}}+\bar{r_{b}}}{2}$$

where r_midpoint_ is the midpoint value of the means of the actual percentages of lesion areas of two adjacent severity classes, $\bar{r_{a}}$ is the mean of the actual percentages of lesion areas corresponding to the lower severity class of *a* in the two adjacent severity classes, and $\bar{r_{b}}$ is the mean of the actual percentages of lesion areas corresponding to the higher severity class of *b* in the two adjacent severity classes.

Method 2: The reference ranges at different levels for all the severity classes were determined by referring to the method for determining the medical reference ranges. In this study, since the actual percentages of the lesion areas in the corresponding whole leaf areas contained in each training set (Train40_R_ or Train30_R_) for each severity class had a normal distribution, the normal distribution method (Sun and Xu, 2014) was used to determine the reference ranges of the actual percentages of the lesion areas for all the severity classes. According to the normal distribution method (Sun and Xu, 2014), for each severity class, the upper and lower limits of the bilateral 100(1–α)% reference range were calculated by using the formula $\bar{r}\pm u_{\alpha /2}s$ , and the unilateral 100(1–α)% reference range was determined by using the formula > $\bar{r}-u_{\alpha }s$ or< $\bar{r}+u_{\alpha }s$ . In the formulas, $\bar{r}$ is the mean of the actual percentages of lesion areas in the corresponding whole leaf areas for a severity class, *s* is the standard deviation of the actual percentages of lesion areas in the corresponding whole leaf areas for the severity class, and u_α_ is the standard normal deviate corresponding to the α value. In this study, based on the values of $\bar{r}$ and *s* of the actual percentages of lesion areas in the corresponding whole leaf areas contained in each training set (Train40_R_ or Train30_R_) for the severity class of *R*, the 90% (α=0.1), 95% (α=0.05), and 99% (α=0.01) reference ranges of the actual percentages of lesion areas corresponding to the severity class of 5%, 10%, 20%, 40%, 60%, 80%, or 100% were determined according to the formulas $\bar{r}\pm 1.64s$ , $\bar{r}\pm 1.96s$ , and $\bar{r}\pm 2.58s$ , respectively. In particular, for the 90% (α=0.1), 95% (α=0.05), and 99% (α=0.01) reference ranges of the severity class of 1%, the lower limits were greater than 0%, and the corresponding upper limits were calculated by using the formulas $\bar{r}+1.28s$ , $\bar{r}+1.64s$ , and $\bar{r}+2.33s$ , respectively.

For a sampling ratio (4:1 or 3:2), if the 90%, 95%, or 99% reference ranges of the actual percentages of lesion areas of adjacent severity classes of wheat stripe rust obtained by using the normal distribution method overlapped, the normal distribution curves of the actual percentage data contained in the training sets of all the severity classes were drawn with the corresponding values of $\bar{r}$ and *s* by using the normal distribution probability density function (*normpdf*) in the software MATLAB 2019b (MathWorks, Natick, MA, USA), and then the intersection point of the normal distribution curves of the actual percentage data contained in the training sets of two adjacent severity classes was obtained by using the function *solve* in the software. The abscissa value of the intersection point was denoted as $\bar{r}$ *a-b* where *a* was the lower one and *b* was the higher one in the two adjacent severity classes. The upper limit of the reference range of the actual percentages of lesion areas corresponding to the severity class of *a* and the lower limit of the reference range of the actual percentages of lesion areas corresponding to the severity class of *b* were determined based on the $\bar{r}$ *a-b* value. Subsequently, the probability of the interval composed of the lower and upper limits of the reference range for a severity class was calculated by using the function *normspec* in the software MATLAB 2019b, and was regarded as the corresponding actual probability of this reference range.

For a sampling ratio, if the normal distribution curve of the actual percentage data contained in the training set of a severity class of *R* had no intersection point with that of the actual percentage data contained in the training set of any adjacent severity class, or had an intersection point, but the $\bar{r}$ *a-b* value lay outside the interval corresponding to the 90%, 95%, or 99% probability of the normal distribution curve of the actual percentage data contained in the training set of the severity class of *R*, the 90%, 95%, or 99% reference range of the actual percentages of lesion areas for the severity class of *R* was estimated according to the formula as described above. If the normal distribution curve of the actual percentage data contained in the training set of a severity class of *R* only had an intersection point with that of the actual percentage data contained in the training set of the lower adjacent severity class, and the $\bar{r}$ *a-b* value lay inside the interval corresponding to the 90%, 95%, or 99% probability of the normal distribution curve of the actual percentage data contained in the training set of the severity class of *R*, the $\bar{r}$ *a-b* value was regarded as the lower limit of the 90%, 95%, or 99% reference range of the actual percentages of lesion areas for the severity class of *R*, then the upper limit of the corresponding reference range was calculated according to the formula as described above, and, subsequently, the probability of the interval composed of the lower and upper limits of the reference range for this severity class calculated by using the function *normspec* in the software MATLAB 2019b was regarded as the corresponding actual probability of this reference range. If the normal distribution curve of the actual percentage data contained in the training set of a severity class of *R* only had an intersection point with that of the actual percentage data contained in the training set of the higher adjacent severity class, and the $\bar{r}$ *a-b* value lay inside the interval corresponding to the 90%, 95%, or 99% probability of the normal distribution curve of the actual percentage data contained in the training set of the severity class of *R*, the $\bar{r}$ *a-b* value was regarded as the upper limit of the 90%, 95%, or 99% reference range of the actual percentages of lesion areas for the severity class of *R*, then the lower limit of the corresponding reference range was calculated according to the formula as described above, and, subsequently, the probability of the interval composed of the lower and upper limits of the reference range for this severity class calculated by using the function *normspec* in the software MATLAB 2019b was regarded as the corresponding actual probability of this reference range. If the normal distribution curve of the actual percentage data contained in the training set of a severity class of *R* had an intersection point with that of the actual percentage data contained in the training set of each of the two adjacent severity classes, and the abscissa values of the two intersection points lay inside the interval corresponding to the 90%, 95%, or 99% probability of the normal distribution curve of the actual percentage data contained in the training set of the severity class of *R*, the two abscissa values were regarded as the lower and upper limits of the 90%, 95%, or 99% reference range of the actual percentages of lesion areas for the severity class of *R*, respectively, and then the probability of the interval composed of the lower and upper limits of the reference range for this severity class calculated by using the function *normspec* in the software MATLAB 2019b was regarded as the corresponding actual probability of this reference range.

In this study, according to the formulas as described above, the estimated 95% reference ranges of the actual percentages of lesion areas for some adjacent severity classes overlapped, and the estimated 99% reference ranges of the actual percentages of lesion areas for all the adjacent severity classes overlapped. Therefore, the 95% or 99% reference ranges of the actual percentages of lesion areas of the adjacent severity classes were obtained according to the method as described above. Although the corresponding probabilities of the reference ranges changed, in a convenient manner, the reference ranges were still called the 95% or 99% reference ranges. In this study, for the two sampling ratios, the calculation methods of the 90%, 95%, and 99% reference ranges of the actual percentages of lesion areas corresponding to each severity class of wheat stripe rust are shown in **Table 1**.

**Table 1:** Calculation methods of the 90%, 95%, and 99% reference ranges of the actual percentages of lesion areas corresponding to each severity class of wheat stripe rust.

Severity class	90% reference range	95% reference range	99% reference range
1%	(0%, $\bar{r}$ +1.28*s*]	(0%, $\bar{r}$ +1.64*s*]	(0%, $\bar{r}$ _1%-5%_]
5%	[ $\bar{r}$ –1.64*s*, $\bar{r}$ +1.64*s*]	[ $\bar{r}$ –1.96*s*, $\bar{r}$ +1.96*s*]	( $\bar{r}$ _1%-5%_, $\bar{r}$ _5%-10%_]
10%	[ $\bar{r}$ –1.64*s*, $\bar{r}$ +1.64*s*]	( $\bar{r}$ _5%-10%_, $\bar{r}$ +1.96*s*]	( $\bar{r}$ _5%-10%_, $\bar{r}$ _10%-20%_]
20%	[ $\bar{r}$ –1.64*s*, $\bar{r}$ +1.64*s*]	[ $\bar{r}$ –1.96*s*, $\bar{r}$ +1.96*s*]	( $\bar{r}$ _10%-20%_, $\bar{r}$ _20%-40%_]
40%	[ $\bar{r}$ –1.64*s*, $\bar{r}$ +1.64*s*]	( $\bar{r}$ _20%-40%_, $\bar{r}$ +1.96*s*]	( $\bar{r}$ _20%-40%_, $\bar{r}$ _40%-60%_]
60%	[ $\bar{r}$ –1.64*s*, $\bar{r}$ +1.64*s*]	[ $\bar{r}$ –1.96*s*, $\bar{r}$ _60%-80%_]	( $\bar{r}$ _40%-60%_, $\bar{r}$ _60%-80%_]
80%	[ $\bar{r}$ –1.64*s*, $\bar{r}$ +1.64*s*]	( $\bar{r}$ _60%-80%_, $\bar{r}$ +1.96*s*]	( $\bar{r}$ _60%-80%_, $\bar{r}$ _80%-100%_]
100%	[ $\bar{r}$ –1.64*s*, $\bar{r}$ +1.64*s*]	[ $\bar{r}$ –1.96*s*, $\bar{r}$ +1.96*s*]	( $\bar{r}$ _80%-100%_, $\bar{r}$ +2.58*s*]

### Severity assessment of each of the acquired diseased wheat leaves with the percentages of lesion areas

According to the midpoint-of-two-adjacent-means-based actual percentage reference ranges and the 90%, 95%, and 99% reference ranges obtained based on the actual percentage data contained in the training set Train40_R_, the severity assessment of each diseased wheat leaf with the actual percentage of lesion area in the area of the corresponding whole diseased leaf contained in the training set Train40_R_ and the testing set Test10_R_ was conducted. In the same way, according to the midpoint-of-two-adjacent-means-based actual percentage reference ranges and the 90%, 95%, and 99% reference ranges obtained based on the actual percentage data contained in the training set Train30_R_, the severity assessment of each diseased wheat leaf with the actual percentage of lesion area in the area of the corresponding whole diseased leaf contained in the training set Train30_R_ and the testing set Test20_R_ was carried out. Then the accuracy of severity assessments of the diseased wheat leaves with the actual percentages of lesion areas contained in each data set of a severity class was calculated by using the following Formula (3).


(3) $$\text{Accuracy}=\frac{n_{\text{p}}}{n}\times 100\%$$

where accuracy is the severity assessment accuracy of the diseased wheat leaves with the actual percentages of lesion areas contained in each data set of a severity class, n_p_ is the number of the diseased leaves correctly assessed, and *n* is the total number of the diseased leaves assessed.

## Results

### The range, mean, and standard deviation of the actual percentage data contained in each of the training sets corresponding to each severity class

Based on the actual percentages of the lesion areas in the areas of the corresponding whole diseased wheat leaves, the range, mean ( $\bar{r}$ ), and standard deviation (*s*) of the actual percentage data contained in each training set (Train40_R_ or Train30_R_) for the severity class of *R* were achieved as shown in **Table 2**. The results showed that the range composed of the minimum and maximum actual percentages for the severity class of *R* (1%, 5%, 10%, 20%, 40%, 60%, 80%, or 100%) obtained based on the actual percentage data contained in Train40_R_ was the same as that obtained based on the actual percentage data contained in Train30_R_. The ranges of actual percentages for the severity classes of 1%, 5%, 10%, 20%, 40%, 60%, 80%, and 100% were [0.06%, 0.78%], [0.85%, 1.64%], [1.73%, 3.29%], [3.65%, 6.31%], [6.76%, 13.88%], [14.22%, 18.43%], [18.90%, 24.15%], and [24.54%, 36.49%], respectively. In Train40_R_ and Train30_R_, the maximum actual percentage of lesion area in the corresponding whole leaf area for the severity class of 100% was 36.49%. Based on the actual percentage data contained in each training set that was called Train40_R_, the means of the actual percentages of lesion areas for the severity classes of 1%, 5%, 10%, 20%, 40%, 60%, 80%, and 100% were 0.40%, 1.27%, 2.50%, 4.92%, 9.89%, 16.61%, 21.23%, and 30.52%, respectively. Based on the actual percentage data contained in each training set that was called Train30_R_, the means of the actual percentages of lesion areas for the severity classes of 1%, 5%, 10%, 20%, 40%, 60%, 80%, and 100% were 0.40%, 1.28%, 2.50%, 4.92%, 9.87%, 16.61%, 21.23%, and 30.53%, respectively. The results showed that there was no obvious difference between the $\bar{r}$ values or the *s* values of the actual percentages of lesion areas in the areas of the corresponding whole diseased leaves contained in Train40_R_ and Train30_R_ at the same severity level (severity class of R). The results demonstrated that for each severity class of wheat stripe rust, there was great difference between the actual percentage of lesion area in the area of a whole diseased leaf and the percentage of the lesion area in the area of the whole diseased leaf corresponding to the severity class in the severity grading standard of the disease as described above.

**Table 2:** Statistics of the actual percentage data of the lesion areas in the areas of the corresponding whole diseased leaves contained in each training set (Train40*_R_* or Train30*_R_*) of the severity class of *R* including the range of actual percentages of lesion areas, mean, and standard deviation.

Data set	Severity class	The range of actual percentages	Mean	Standard deviation
Train40_1%_	1%	[0.06%, 0. 78%]	0.40%	0.19%
Train40_5%_	5%	[0.85%, 1.64%]	1.27%	0.23%
Train40_10%_	10%	[1.73%, 3.29%]	2.50%	0.42%
Train40_20%_	20%	[3.65%, 6.31%]	4.92%	0.78%
Train40_40%_	40%	[6.76%, 13.88%]	9.89%	1.97%
Train40_60%_	60%	[14.22%, 18.43%]	16.61%	1.21%
Train40_80%_	80%	[18.90%, 24.15%]	21.23%	1.41%
Train40_100%_	100%	[24.54%, 36.49%]	30.52%	3.19%
Train30_1%_	1%	[0.06%, 0.78%]	0.40%	0.19%
Train30_5%_	5%	[0.85%, 1.64%]	1.28%	0.23%
Train30_10%_	10%	[1.73%, 3.29%]	2.50%	0.43%
Train30_20%_	20%	[3.65%, 6.31%]	4.92%	0.78%
Train30_40%_	40%	[6.76%, 13.88%]	9.87%	1.95%
Train30_60%_	60%	[14.22%, 18.43%]	16.61%	1.20%
Train30_80%_	80%	[18.90%, 24.15%]	21.23%	1.43%
Train30_100%_	100%	[24.54%, 36.49%]	30.53%	3.21%

### The determined reference ranges of the actual percentages of lesion areas in the corresponding whole leaf areas for all the severity classes

For each sampling ratio (4:1 or 3:2), a total of four sets of reference ranges of the actual percentages of lesion areas in the corresponding whole leaf areas for all the severity classes of wheat stripe rust, including the midpoint-of-two-adjacent-means-based actual percentage reference ranges, the 90% reference ranges, the 95% reference ranges, and the 99% reference ranges, were determined as shown in **Table 3**.

**Table 3:** The determined reference ranges of the actual percentages of lesion areas in the corresponding whole leaf areas for all the severity classes based on the actual percentage data in the training sets Train40*_R_* and Train30*_R_* and the actual probabilities for the corresponding 90%, 95%, and 99% reference ranges.

Data set	Severity class	Midpoint-of-two-adjacent-means-based actual percentage reference range	90% reference range	Actual probability corresponding to the 90% reference range	95% reference range	Actual probability corresponding to the 95% reference range	99% reference range	Actual probability corresponding to the 99% reference range
Train40_1%_	1%	(0, 0.84%]	(0%,0.64%]	90%	(0%, 0.71%]	95%	(0%, 0.80%]	96.47%
Train40_5%_	5%	(0.84%, 1.89%]	[0.89%, 1.65%]	90%	[0.82%, 1.72%]	95%	(0.80%, 1.75%]	96.11%
Train40_10%_	10%	(1.89%, 3.71%]	[1.81%, 3.19%]	90%	(1.75%, 3.32%]	93.75%	(1.75%, 3.43%]	94.95%
Train40_20%_	20%	(3.71%, 7.41%]	[3.64%, 6.20%]	90%	[3.39%, 6.45%]	95%	(3.43%, 6.60%]	95.63%
Train40_40%_	40%	(7.41%, 13.25%]	[6.66%,13.12%]	90%	(6.60%,13.75%]	92.75%	(6.60%,13.88%]	93.11%
Train40_60%_	60%	(13.25%, 18.92%]	[14.63%, 18.59%]	90%	[14.24%, 18.80%]	93.98%	(13.88%, 18.80%]	95.28%
Train40_80%_	80%	(18.92%, 25.88%]	[18.92%, 23.54%]	90%	(18.80%, 23.99%]	93.24%	(18.80%, 24.46%]	94.66%
Train40_100%_	100%	(25.88%, 100%]	[25.29%, 35.75%]	90%	[24.27%, 36.77%]	95%	(24.46%, 38.75%]	96.63%
Train30_1%_	1%	(0, 0.84%]	(0%,0.64%]	90%	(0%, 0.71%]	95%	(0%, 0.81%]	96.69%
Train30_5%_	5%	(0.84%, 1.89%]	[0.90%, 1.66%]	90%	[0.83%, 1.73%]	95%	(0.81%, 1.75%]	95.90%
Train30_10%_	10%	(1.89%, 3.71%]	[1.79%, 3.21%]	90%	(1.75%, 3.34%]	93.41%	(1.75%, 3.44%]	94.50%
Train30_20%_	20%	(3.71%, 7.40%]	[3.64%, 6.20%]	90%	[3.39%, 6.45%]	95%	(3.44%, 6.60%]	95.55%
Train30_40%_	40%	(7.40%, 13.24%]	[6.67%, 13.07%]	90%	(6.60%, 13.69%]	92.82%	(6.60%, 13.88%]	93.33%
Train30_60%_	60%	(13.24%, 18.92%]	[14.64%, 18.58%]	90%	[14.26%, 18.78%]	93.96%	(13.88%, 18.78%]	95.33%
Train30_80%_	80%	(18.92%, 25.88%]	[18.88%, 23.58%]	90%	(18.78%, 24.03%]	93.16%	(18.78%, 24.48%]	94.51%
Train30_100%_	100%	(25.88%, 100%]	[25.27%, 35.79%]	90%	[24.24%, 36.82%]	95%	(24.48%, 38.81%]	96.53%

Based on the actual percentage data contained in the training sets Train40_R_ and Train30_R_, the determined midpoint-of-two-adjacent-means-based actual percentage reference ranges corresponding to the severity class of *R*, as shown in **Table 3**, were obtained by taking the values of r_midpoint_ of the means of the actual percentages of lesion areas in the corresponding whole leaf areas of two adjacent severity classes as the demarcation points. Based on the actual percentage data contained in each training set that was called Train40_R_, the midpoint-of-two-adjacent-means-based actual percentage reference ranges for the severity classes of 1%, 5%, 10%, 20%, 40%, 60%, 80%, and 100% were (0, 0.84%], (0.84%, 1.89%], (1.89%, 3.71%], (3.71%, 7.41%], (7.41%, 13.25%], (13.25%, 18.92%], (18.92%, 25.88%], and (25.88%, 100%], respectively. Based on the actual percentage data contained in each training set that was called Train30_R_, the midpoint-of-two-adjacent-means-based actual percentage reference ranges for the severity classes of 1%, 5%, 10%, 20%, 40%, 60%, 80%, and 100% were (0, 0.84%], (0.84%, 1.89%], (1.89%, 3.71%], (3.71%, 7.40%], (7.40%, 13.24%], (13.24%, 18.92%], (18.92%, 25.88%], and (25.88%, 100%], respectively. The results showed that for the severity class of *R*, the midpoint-of-two-adjacent-means-based actual percentage reference range obtained based on the actual percentage data in Train40_R_ was similar to that obtained based on the actual percentage data in Train30_R_. The midpoint-of-two-adjacent-means-based actual percentage reference range for each severity class obtained based on the actual percentage data contained in each corresponding training set by using the method as described above, had relatively large difference with the range (as shown in **Table 2**) composed of the minimum and maximum actual percentages for the corresponding severity class.

For the sampling ratio of 4:1, based on the actual percentage data contained in each training set that was called Train40_R_, the obtained 90%, 95%, and 99% reference ranges of the actual percentages of lesion areas corresponding to each severity class and the actual probabilities for the corresponding reference ranges are shown in **Table 3**. For the sampling ratio of 3:2, based on the actual percentage data contained in each training set that was called Train30_R_, the obtained 90%, 95%, and 99% reference ranges of the actual percentages of lesion areas corresponding to each severity class and the actual probabilities for the corresponding reference ranges are also shown in **Table 3**. On the whole, the obtained 90%, 95%, or 99% reference range for the severity class of *R* based on the actual percentage data in Train40_R_ had small difference with the corresponding 90%, 95%, or 99% reference range for the severity class of *R* based on the actual percentage data in Train30_R_. For the obtained 90%, 95%, and 99% reference ranges based on the actual percentage data contained in each training set of a severity class of *R* (1%, 5%, 10%, 20%, 40%, 60%, 80%, or 100%), the 95% and 99% reference ranges of the severity class hade small difference, but both of them had relatively large differences with the 90% reference range of the corresponding severity class.

The results indicated that based on the actual percentages of the lesion areas in the corresponding whole leaf areas contained in each training set (Train40_R_ or Train30_R_), the 90%, 95%, and 99% reference ranges of the actual percentages of lesion areas corresponding to each severity class obtained by using the normal distribution method had relatively obvious differences with the obtained midpoint-of-two-adjacent-means-based actual percentage reference range for the corresponding severity class. Moreover, the obtained 90%, 95%, and 99% reference ranges of the actual percentages of lesion areas for the severity class had certain differences with the range (as shown in **Table 2**) composed of the minimum and maximum actual percentages for the corresponding severity class.

### Severity assessment results for the acquired diseased wheat leaves with the actual percentages of lesion areas according to the determined reference ranges

For the sampling ratio of 4:1, according to the determined reference ranges based on the actual percentage data contained in the training sets for all severity classes of wheat stripe rust, including the midpoint-of-two-adjacent-means-based actual percentage reference ranges and the 90%, 95%, and 99% reference ranges of the actual percentages of lesion areas, the results of severity assessment of each diseased wheat leaf contained in the corresponding training sets are shown in **Table 4**. For the sampling ratio of 3:2, according to the midpoint-of-two-adjacent-means-based actual percentage reference range and the 90%, 95%, and 99% reference ranges of the actual percentages of lesion areas determined based on the actual percentage data contained in each training set that was called Train30_R_, the results of severity assessment of each diseased wheat leaf contained in the corresponding training set are also shown in **Table 4**. The results demonstrated that satisfactory assessment accuracies for the training sets could be achieved by using each set of the determined reference ranges for all the severity classes of wheat stripe rust, and that the assessment accuracy for each training set (Train40_R_ or Train30_R_) was not lower than 85%. For the sampling ratio of 4:1, according to the midpoint-of-two-adjacent-means-based actual percentage reference ranges and the 90%, 95%, and 99% reference ranges of the actual percentages of lesion areas based on the actual percentage data contained in the training sets for all the severity classes, among the assessment accuracies for all the corresponding training sets, the lowest accuracies were 85.00%, 87.50%, 95.00%, and 95.00%, respectively. For the sampling ratio of 3:2, according to the midpoint-of-two-adjacent-means-based actual percentage reference ranges and the 90%, 95%, and 99% reference ranges of the actual percentages of lesion areas based on the actual percentage data contained in the training sets for all the severity classes, the lowest accuracies were 86.67%, 90.00%, 96.67%, and 96.67%, respectively, among the assessment accuracies for all the corresponding training sets. On the whole, for the sampling ratio of 4:1 or 3:2, the severity assessment results of all the diseased wheat leaves contained in the training set of a severity class according to the 90%, 95%, and 99% reference ranges of the actual percentages of lesion areas for the corresponding severity class indicated that the 99% reference range had the best assessment performance and that the assessment performance of the 95% reference range ranked second. Furthermore, the severity assessment results of all the diseased wheat leaves contained in the training set of a severity class according to the midpoint-of-two-adjacent-means-based actual percentage reference range and the 90% reference ranges of the actual percentages of lesion areas for the corresponding severity class indicated that the two reference ranges had the similar assessment performances.

**Table 4:** Severity assessment results of the diseased wheat leaves with the actual percentages of lesion areas contained in each training set of all the severity classes of wheat stripe rust according to the determined reference ranges.

Severity class	Training set	Assessment accuracy based on the midpoint-of-two-adjacent-means-based actual percentage reference range	Assessment accuracy based on the 90% reference range	Assessment accuracy based on the 95% reference range	Assessment accuracy based on the 99% reference range
1%	Train40_1%_	100.00%	95.00%	97.50%	100.00%
	Train30_1%_	100.00%	93.33%	96.67%	100.00%
5%	Train40_5%_	100.00%	95.00%	100.00%	100.00%
	Train30_5%_	100.00%	93.33%	100.00%	100.00%
10%	Train40_10%_	87.50%	90.00%	100.00%	100.00%
	Train30_10%_	86.67%	90.00%	96.67%	96.67%
20%	Train40_20%_	97.50%	95.00%	100.00%	100.00%
	Train30_20%_	96.67%	96.67%	100.00%	100.00%
40%	Train40_40%_	85.00%	95.00%	95.00%	100.00%
	Train30_40%_	86.67%	96.67%	96.67%	96.67%
60%	Train40_60%_	100.00%	92.50%	97.50%	95.00%
	Train30_60%_	100.00%	93.33%	96.67%	100.00%
80%	Train40_80%_	97.50%	90.00%	97.50%	100.00%
	Train30_80%_	96.67%	93.33%	96.67%	100.00%
100%	Train40_100%_	90.00%	87.50%	100.00%	100.00%
	Train30_100%_	90.00%	90.00%	100.00%	100.00%

According to the midpoint-of-two-adjacent-means-based actual percentage reference range and the 90%, 95%, and 99% reference ranges of the actual percentages of lesion areas determined based on the actual percentage data in the training set Train40_R_ for a severity classes of *R*, the results of severity assessments of the diseased leaves contained in the corresponding testing set Test10_R_ are shown in **Table 5**. According to the midpoint-of-two-adjacent-means-based actual percentage reference range and the 90%, 95%, and 99% reference ranges of the actual percentages of lesion areas determined based on the actual percentage data in the training set Train30_R_ for a severity class of *R*, the results of severity assessments of the diseased leaves contained in the corresponding testing set Test20_R_ are also shown in **Table 5**. The results demonstrated that satisfactory assessment accuracies for the testing sets could be achieved according to the midpoint-of-two-adjacent-means-based actual percentage reference ranges and the 90%, 95%, and 99% reference ranges of the actual percentages of lesion areas in the corresponding whole leaf areas for all the severity classes of wheat stripe rust, and that the assessment accuracy for each testing set (Test10_R_ or Test20_R_) was not lower than 85%. For the sampling ratio of 4:1, according to the midpoint-of-two-adjacent-means-based actual percentage reference ranges and the 90% reference ranges of the actual percentages of lesion areas based on the actual percentage data contained in the training sets for all the severity classes, the lowest accuracies were both 90.00% among the assessment accuracies for all the corresponding testing sets; according to the 95% and 99% reference ranges of the actual percentages of lesion areas based on the actual percentage data contained in the training sets for all the severity classes, the assessment accuracies for all the corresponding testing sets were 100.00%. For the sampling ratio of 3:2, according to the midpoint-of-two-adjacent-means-based actual percentage reference ranges and the 90%, 95%, and 99% reference ranges of the actual percentages of lesion areas based on the actual percentage data contained in the training sets for all the severity classes, the lowest accuracies were 85.00%, 85.00%, 95.00%, and 95.00%, respectively, among the assessment accuracies for all the corresponding testing sets. Overall, for the sampling ratio of 4:1 or 3:2, the severity assessment results of all the diseased wheat leaves contained in the testing set of a severity class according to the midpoint-of-two-adjacent-means-based actual percentage reference range and the 90%, 95%, and 99% reference ranges of the actual percentages of lesion areas for the corresponding severity class demonstrated that the 99% reference range had the best assessment performance, the assessment performance of the 95% reference range ranked second, that of the 90% reference range ranked third, and that of the midpoint-of-two-adjacent-means-based actual percentage reference range ranked last. The assessment performance of the midpoint-of-two-adjacent-means-based actual percentage reference range, the 90% reference range, the 95% reference range, or the 99% reference range determined based on the actual percentage data in the training set Train40_R_ for a severity class of *R* when the reference range was used to assess all the diseased wheat leaves contained in the testing set Test10_R_, was better than that of the corresponding reference range determined based on the actual percentage data in the training set Train30_R_ for the severity class of *R* when it was used to assess all the diseased wheat leaves contained in the testing set Test20_R_.

**Table 5:** Severity assessment results of the diseased wheat leaves with the actual percentages of lesion areas contained in each testing set of all the severity classes of wheat stripe rust according to the determined reference ranges.

Severity class	Testing set	Assessment accuracy based on the midpoint-of-two-adjacent-means-based actual percentage reference range	Assessment accuracy based on the 90% reference range	Assessment accuracy based on the 95% reference range	Assessment accuracy based on the 99% reference range
1%	Test10_1%_	100.00%	90.00%	100.00%	100.00%
	Test20_1%_	100.00%	95.00%	100.00%	100.00%
5%	Test10_5%_	100.00%	90.00%	100.00%	100.00%
	Test20_5%_	100.00%	95.00%	100.00%	100.00%
10%	Test10_10%_	90.00%	90.00%	100.00%	100.00%
	Test20_10%_	90.00%	90.00%	95.00%	95.00%
20%	Test10_20%_	100.00%	100.00%	100.00%	100.00%
	Test20_20%_	100.00%	95.00%	100.00%	100.00%
40%	Test10_40%_	90.00%	100.00%	100.00%	100.00%
	Test20_40%_	85.00%	95.00%	95.00%	100.00%
60%	Test10_60%_	100.00%	90.00%	100.00%	100.00%
	Test20_60%_	100.00%	90.00%	100.00%	100.00%
80%	Test10_80%_	100.00%	90.00%	100.00%	100.00%
	Test20_80%_	100.00%	90.00%	100.00%	100.00%
100%	Test10_100%_	90.00%	90.00%	100.00%	100.00%
	Test20_100%_	90.00%	85.00%	100.00%	100.00%

The results demonstrated that according to the two developed methods based on the reference ranges of the percentages of lesion areas for severity assessment of wheat stripe rust in this study, high accuracy can be obtained in the severity assessments of the diseased leaves, indicating that the two methods were suitable for the severity assessment of the disease. In the practical applications, the midpoint-of-two-adjacent-means-based actual percentage reference ranges can be used to carry out severity assessment of wheat stripe rust, or according to the accuracy requirements for the severity assessment results, a set of reference ranges can be selected for severity assessment of the disease from the 90%, 95%, and 99% reference ranges of the actual percentages of lesion areas corresponding to all the severity classes.

## Discussion

In this study, two new methods for severity assessment of wheat stripe rust were proposed based on the actual percentages of lesion areas in the areas of the corresponding whole wheat leaves. The main characteristics of the two proposed methods are shown in **Table 6**. By using the methods, the suitable reference range selected from the midpoint-of-two-adjacent-means-based actual percentage reference ranges and the 90%, 95%, and 99% reference ranges of the actual percentages of lesion areas corresponding to all the severity classes of wheat stripe rust can be directly used to assess the severity of each diseased wheat leaf with the actual percentage of lesion area in the area of the corresponding whole leaf. The two methods are simple, easy-to-operate, rapid, and accurate. The methods are applicable to all plant diseases for which the severity is classified according to the ratio of lesion area to the area of the corresponding whole diseased plant unit. The method for determination of the midpoint-of-two-adjacent-means-based actual percentage reference ranges corresponding to all the disease severity classes and the method for determination of the 90%, 95%, and 99% reference ranges of the actual percentages of lesion areas corresponding to all the disease severity classes, are provided for severity assessments of plant diseases. The basis of the two methods for disease severity assessment is very intuitive and in line with human psychological cognitive habits. The two methods are very convenient for practical operations and can improve the accuracy of plant disease severity assessment, resulting in more reliable plant disease information for diseased plant phenotyping, disease prediction and forecast, and disease management. The two methods are conducive to solve the classification difficulties in assessing the severity of plant diseases. Especially, during severity assessments of plant diseases according to the ratio of lesion area to the area of the whole diseased plant unit, for some plant diseases such as wheat stripe rust and wheat leaf rust, the ratio of the lesion area to the area of the whole diseased plant unit corresponding to a severity class in the disease severity grading standard is not the actual ratio of the lesion area to the area of the whole diseased plant unit, which can induce great errors or complete errors in the severity assessment results. This problem was well solved in this study, which provided a basis and methodological reference for accurate severity assessments of plant diseases and was of great significance for survey, monitoring, prediction, and control of plant diseases.

**Table 6:** The main characteristics of the two proposed methods for determining the reference ranges of the actual percentages of lesion areas in the corresponding whole leaf areas for all the severity classes of wheat stripe rust.

Method	Devices used to acquire images	Image assessment method	Mathematical algorithms used for image evaluation	Statistical evaluation of the data obtained from the images	The determined reference range	Reliability of the obtained results
Method 1	Nikon D700 digital camera, HUAWEI P30 smartphone, and iPhone 6S smartphone.	Manual image segmentation and pixel statistics in the Adobe Photoshop software.	Calculation of the actual percentage (*r*) of the lesion area in the area of the whole diseased leaf by using the formula: $r=\frac{A_{\text{d}}}{A_{\text{l}}}\times 100\%$ where *A*_d_ is the pixel number of the lesion region/regions in the diseased leaf image, and *A*_l_ is the pixel number of the whole leaf region in the diseased leaf image.	Mean, standard deviation, the midpoint value of the means of the actual percentages of lesion areas of two adjacent severity classes.	The midpoint-of-two-adjacent-means-based actual percentage reference ranges.	Assessment accuracy≥85.00%.
Method 2				Mean, standard deviation, normal distribution test, normal distribution method for determining the bilateral 100(1–α)% reference ranges and the unilateral 100(1–α)% reference ranges by combining the normal distribution probability density function (*normpdf*) and the functions *solve* and *normspec* in the MATLAB software.	The 90%, 95%, and 99% reference ranges of the actual percentages of lesion areas.	Assessment accuracy≥85.00% for the 90% reference ranges, and ≥95.00% for the 95% and 99% reference ranges.

In this study, 50 single diseased wheat leaf images for each severity class of wheat stripe rust were acquired and the actual percentages of the lesion areas in the corresponding whole leaf areas were obtained. For each severity class of the disease, the training sets and testing sets were constructed by using the system sampling method with two sampling ratios of 4:1 and 3:2. The representative values of the actual percentages of lesion areas corresponding to each severity class for the two sampling ratios had no obvious difference. For the sampling ratio of 4:1 or 3:2, high assessment accuracies for the training set and testing set were achieved according to the midpoint-of-two-adjacent-means-based actual percentage reference range and the 90%, 95%, and 99% reference ranges of the actual percentages of lesion areas in the corresponding whole leaf areas for each severity class. In comparison, by and large, the assessment performance of the midpoint-of-two-adjacent-means-based actual percentage reference range, the 90% reference range, the 95% reference range, or the 99% reference range determined based on the actual percentage data in the training set Train40_R_ constructed by using the sampling ratio of 4:1 was better than that of the corresponding reference range determined based on the actual percentage data in the training set Train30_R_ constructed by using the sampling ratio of 3:2. If more images of the single diseased wheat leaves for each severity class of the disease can be acquired, the more ideal reference ranges for each severity class may be obtained by using the proposed methods in this study, and thus the better severity assessment results may be achieved.

The results obtained in this study showed that the actual percentage of lesion area in the area of a whole diseased leaf corresponding to each severity class of wheat stripe rust had great difference with the percentage of the lesion area in the area of the whole diseased leaf corresponding to the severity class in the disease severity grading standard, which is consistent with the results obtained by Shang et al. (1990). The maximum actual percentage of the lesion area in the area of the whole diseased wheat leaf with the most severe disease symptom among the collected diseased wheat leaves obtained by using image processing technology in this study was 36.49%, and it was higher than the maximum actual uredinium coverage rate of 35% obtained by Shang et al. (1990) via actual measurement of the selected wheat leaf with the most severe disease symptom. The maximum actual percentage of lesion area obtained in this study should be more close to the true value of the percentage of the lesion area in the area of the whole diseased wheat leaf with the most severe disease symptom of wheat stripe rust. Therefore, it is believed that in this study, whether the sampling ratio was 4:1 or 3:2, each set of the determined midpoint-of-two-adjacent-means-based actual percentage reference ranges could cover all possible actual percentages of lesion areas in the corresponding whole leaf areas for all the severity classes of wheat stripe rust, and each set of the determined 99% reference ranges of the actual percentages of lesion areas could basically cover all possible actual percentages of lesion areas in the corresponding whole leaf areas for all the severity classes of the disease. Each set of the determined 90% reference ranges of the actual percentages of lesion areas or each set of the determined 95% reference ranges of the actual percentages of lesion areas could basically meet the accuracy requirements of severity assessment of wheat stripe rust, although there were gaps between the reference ranges of some adjacent severity classes. If necessary, an actual percentage of lesion area falling into a gap can be assessed as the severity class corresponding to the nearest reference range according to the nearest percent estimate principle (by taking the value of the midpoint of the gap as the demarcation point). In practice, a set of the determined midpoint-of-two-adjacent-means-based actual percentage reference ranges or a set of the determined 99% reference ranges of the actual percentages of lesion areas can be selected, aiming to use a set of reference ranges that can cover all possible actual percentages of lesion areas in the corresponding whole leaf areas for all the severity classes to carry out disease severity assessment.

In this study, when determining the reference ranges for disease severity assessment, the standard deviation was directly used, rather than the standard error. The difference between reference range and confidence interval should be paid attention to. The confidence interval is the estimation interval of a population parameter obtained by the sample statistics. When determining a confidence interval, the standard error is directly used, rather than the standard deviation. In this study, based on the constructed training sets, the 90%, 95%, and 99% confidence intervals were also estimated (as shown in **Supplementary Table 1**), and then by using these different confidence intervals as the reference ranges, the severity assessments of the diseased wheat leaves with the actual percentages of lesion areas in the areas of the whole diseased leaves contained in the corresponding training sets and testing sets were conducted, but the obtained assessment accuracies (as shown in **Supplementary Table 2**) were not high. The results indicated that in order to obtain satisfactory severity assessment results, the reference ranges for disease severity assessments should be determined by using the methods proposed in this study.

In the field of medicine, the reference ranges of the normal values of various medical indicators are the normal fluctuation ranges of the corresponding indicators of the vast majority of normal people, and they are used to evaluate whether the measured corresponding indicators are normal and can provide a basis for disease diagnosis, health assessment, and disease treatment (Horn and Pesce, 2003; Sun and Xu, 2014; Haeckel et al., 2021; Yang et al., 2022). There are many methods to determine the medical reference ranges (Horn and Pesce, 2003; Sun and Xu, 2014; Haeckel et al., 2021; Yang et al., 2022). A medical reference range is usually determined by using normal distribution method or percentile method, mainly depending on whether the related data conform to a normal distribution (Horn and Pesce, 2003; Sun and Xu, 2014; Haeckel et al., 2021). When the related data of the corresponding indicator conform to a normal distribution or can be transformed into a normal distribution via data transformation, normal distribution method can be used to estimate the reference range, otherwise, when the data do not conform to a normal distribution, percentile method can be used (Horn and Pesce, 2003; Sun and Xu, 2014; Haeckel et al., 2021). In this study, the reference ranges at different levels (90%, 95%, and 99%) of the actual percentages of lesion areas corresponding to each severity class of wheat stripe rust were estimated by referring to the method for determining medical reference ranges, and the determined 90%, 95%, and 99% reference ranges of the actual percentages of lesion areas corresponding to each severity class can be considered as the fluctuation ranges of the actual percentages of lesion areas of 90%, 95%, and 99% of diseased leaves of the corresponding severity class, respectively. In this study, 50 actual percentages of the lesion areas in the areas of the corresponding whole leaves of each severity class of wheat stripe rust had a normal distribution, and the actual percentages of lesion areas contained in each constructed training set conformed to a normal distribution, so the normal distribution method was used to determine the 90%, 95%, and 99% reference ranges of the actual percentages of lesion areas corresponding to each severity class of the disease. When the method proposed in this study is used to determine the reference ranges at different levels of the actual percentages of lesion areas corresponding to each severity class of a plant disease, the determination method of the reference ranges should be modified or changed if the actual percentage data do not conform to a normal distribution. The actual percentage data can be transformed into a normal distribution through data transformation and then the normal distribution method can be used to determine the reference ranges, or the other methods including the percentile method can be used (Horn and Pesce, 2003; Sun and Xu, 2014; Haeckel et al., 2021).

In this study, the images of the single diseased wheat leaves of all the severity classes of wheat stripe rust were acquired by using digital camera and smartphones, the segmented leaf images and the segmented lesion images were obtained by using manual image segmentation method in the Adobe Photoshop 2022 software, then the numbers of the whole leaf region pixels and lesion region pixels of each single wheat leaf were achieved by viewing the histogram panel in the software, and subsequently the actual percentage of the lesion area in the area of each whole diseased leaf was calculated for further data processing. In terms of obtaining the actual ratios of lesion areas to the areas of the corresponding whole diseased plant units, in addition to the method of obtaining the actual percentages of lesion areas in the areas of the corresponding whole diseased wheat leaves used in this study, automatic image processing methods can be used to carry out disease image segmentation and obtain the actual ratios of lesion areas to the areas of the corresponding whole diseased plant units by programming or by using the developed software and packages such as APS Assess (Lamari, 2008), ImageJ (Schneider et al., 2012), Leaf Doctor (Pethybridge and Nelson, 2015), and the pliman package (Olivoto et al., 2022), and in some situations, the graph paper method (Li et al., 2011) and the paper-weighing method (Li et al., 2011) can be used to achieve the actual ratios of lesion areas. After obtaining the actual ratios of lesion areas to the areas of the corresponding whole diseased plant units for a plant disease, the reference ranges corresponding to all the plant disease severity classes can be determined according to the methods proposed in this study and then can be used to carry out the disease severity assessment, or the disease severity assessment can be directly carried out according to the severity grading standard established based on the actual ratios of lesion areas to the areas of the corresponding whole diseased plant units.

At present, in the studies and practical applications of plant disease severity assessment based on image processing technology, the severity classes are determined according to the ratios of segmented lesion areas to the areas of the corresponding whole diseased plant units (Chen et al., 2008; Guan et al., 2010; Li et al., 2011; Barbedo, 2014; Shrivastava et al., 2015; Jiang et al., 2021) or identified by using the established recognition models based on the extracted image features (Bai et al., 2011; Wang et al., 2017; Bao et al., 2018; Bao et al., 2021). However, in the reported studies on the severity assessment of some plant diseases such as wheat stripe rust based on the ratios of lesion areas to the areas of the corresponding whole disease plant units, it was not taken into account that the actual ratios of lesion areas for each disease severity class are obviously lower than the corresponding ratios of lesion areas of the estimated severity class according to the corresponding severity grading standard. The previous understanding of plant disease severity in the plant disease severity assessment can be corrected by using the two methods for disease severity assessment proposed in this study, which will greatly improve the accuracy of plant disease severity assessment and the reliability of plant disease monitoring and early warning information based on image processing technology. Some basis and research ideas for the realization of automatic assessment of plant disease severity based on image processing technology were provided in this study, which is conducive to the automation and intellectualization of plant disease severity assessment and is helpful to improve the levels of disease survey, disease monitoring and early warning, and disease management, thus providing more reliable supports for diseased plant phenotyping, disease monitoring, disease prediction and forecast, and disease control strategy making.

## Conclusion

Two new methods were developed based on the reference ranges of the actual percentages of lesion areas for severity assessment of wheat stripe rust in this study. Based on the acquired single diseased wheat leaf images of all the severity classes of the disease, the actual percentage of the lesion area in the area of the corresponding whole diseased leaf for each disease image was obtained by using image processing technology, the training sets and testing sets were constructed by using the system sampling method with two sampling ratios, then the methods were developed for determination of the midpoint-of-two-adjacent-means-based actual percentage reference ranges and the reference ranges of the actual percentages of lesion areas at different levels for all the severity classes, and simultaneously the corresponding detailed reference ranges were provided. The satisfactory assessment accuracies for the training and testing sets were achieved according to the determined midpoint-of-two-adjacent-means-based actual percentage reference ranges and the estimated 90%, 95%, and 99% reference ranges of the actual percentages of lesion areas for all the severity classes. In this study, two simple and practical methods were provided for the severity assessment of wheat stripe rust and a reference was provided for accurate severity assessments of plant diseases.

## Data availability statement

The original contributions presented in the study are included in the article/**Supplementary Material**. Further inquiries can be directed to the corresponding author.

## Author contributions

HGW contributed conception of the study and designed the experiments. QJ and HLW performed the experiments. QJ and HGW analyzed the data. QJ and HGW wrote the draft of the manuscript. All authors contributed to manuscript revision, read and approved the final version of the manuscript. All authors contributed to the article and approved the submitted version.

## Funding

This work was supported by the National Key Basic Research Program of China (Grant No. 2021YFD1401001 and 2018YFD0200402) and the National Natural Science Foundation of China (Grant No. 32072357).

## Conflict of interest

The authors declare that the research was conducted in the absence of any commercial or financial relationships that could be construed as a potential conflict of interest.

## Publisher’s note

All claims expressed in this article are solely those of the authors and do not necessarily represent those of their affiliated organizations, or those of the publisher, the editors and the reviewers. Any product that may be evaluated in this article, or claim that may be made by its manufacturer, is not guaranteed or endorsed by the publisher.
