# Supplementary material for: Two new methods for severity assessment of wheat stripe rust caused by Puccinia striiformis f. sp. tritici
Source: Front Plant Sci. 2022 Oct 3;13:1002627. doi: 10.3389/fpls.2022.1002627 (PMC9808611; doi:10.3389/fpls.2022.1002627)
Supplement: Supplementary file 1 [file DataSheet_1.docx]

**Supplementary Tables:**

**Supplementary Table 1.** The confidence intervals of the actual percentages of lesion areas in the corresponding whole leaf areas for all the severity classes based on the actual percentage data contained in the training sets Train40*_R_* and Train30*_R_*.

| Data set | Severity class | 90% confidence interval | 95% confidence interval | 99% confidence interval |
| --- | --- | --- | --- | --- |
| Train40_1%_ | 1% | [0.35%, 0.45%] | [0.34%, 0.46%] | [0.32%, 0.48%] |
| Train40_5%_ | 5% | [1.21%, 1.34%] | [1.20%, 1.35%] | [1.18%, 1.37%] |
| Train40_10%_ | 10% | [2.39%, 2.62%] | [2.37%, 2.64%] | [2.32%, 2.68%] |
| Train40_20%_ | 20% | [4.71%, 5.13%] | [4.67%, 5.17%] | [4.59%, 5.25%] |
| Train40_40%_ | 40% | [9.36%, 10.42%] | [9.26%, 10.52%] | [9.04%, 10.74%] |
| Train40_60%_ | 60% | [16.29%, 16.93%] | [16.23%, 17.00%] | [16.10%, 17.13%] |
| Train40_80%_ | 80% | [20.85%, 21.60%] | [20.78%, 21.68%] | [20.62%, 21.83%] |
| Train40_100%_ | 100% | [29.67%, 31.37%] | [29.50%, 31.54%] | [29.15%, 31.88%] |
| Train30_1%_ | 1% | [0.34%, 0.46%] | [0.33%, 0.47%] | [0.31%, 0.50%] |
| Train30_5%_ | 5% | [1.20%, 1.35%] | [1.19%, 1.36%] | [1.16%, 1.39%] |
| Train30_10%_ | 10% | [2.37%, 2.63%] | [2.34%, 2.66%] | [2.29%, 2.72%] |
| Train30_20%_ | 20% | [4.68%, 5.16%] | [4.63%, 5.21%] | [4.53%, 5.31%] |
| Train30_40%_ | 40% | [9.26%, 10.47%] | [9.14%, 10.59%] | [8.89%, 10.85%] |
| Train30_60%_ | 60% | [16.24%, 16.98%] | [16.16%, 17.06%] | [16.00%, 17.21%] |
| Train30_80%_ | 80% | [20.79%, 21.68%] | [20.70%, 21.77%] | [20.51%, 21.95%] |
| Train30_100%_ | 100% | [29.54%, 31.53%] | [29.33%, 31.73%] | [28.92%, 32.15%] |

**Supplementary Table 2.** Severity assessment results of the diseased wheat leaves with the actual percentages of lesion areas contained in each training set and testing set of all the severity classes of wheat stripe rust according to the confidence intervals.

| Severity class | Data set | Assessment accuracy based on the 90% confidence interval | Assessment accuracy based on the 95% confidence interval | Assessment accuracy based on the 99% confidence interval |
| --- | --- | --- | --- | --- |
| 1% | Train40_1%_ | 55.00% | 55.00% | 57.50% |
|  | Train30_1%_ | 56.67% | 56.67% | 63.33% |
| 5% | Train40_5%_ | 22.50% | 22.50% | 25.00% |
|  | Train30_5%_ | 23.33% | 23.33% | 30.00% |
| 10% | Train40_10%_ | 20.00% | 20.00% | 25.00% |
|  | Train30_10%_ | 23.33% | 23.33% | 36.67% |
| 20% | Train40_20%_ | 15.00% | 20.00% | 27.50% |
|  | Train30_20%_ | 20.00% | 20.00% | 30.00% |
| 40% | Train40_40%_ | 20.00% | 22.50% | 35.00% |
|  | Train30_40%_ | 20.00% | 26.67% | 36.67% |
| 60% | Train40_60%_ | 15.00% | 20.00% | 25.00% |
|  | Train30_60%_ | 16.67% | 26.67% | 33.33% |
| 80% | Train40_80%_ | 20.00% | 27.50% | 30.00% |
|  | Train30_80%_ | 26.67% | 26.67% | 40.00% |
| 100% | Train40_100%_ | 25.00% | 30.00% | 32.50% |
|  | Train30_100%_ | 26.67% | 33.33% | 40.00% |
| 1% | Test10_1%_ | 50.00% | 60.00% | 60.00% |
|  | Test20_1%_ | 55.00% | 55.00% | 60.00% |
| 5% | Test10_5%_ | 20.00% | 20.00% | 20.00% |
|  | Test20_5%_ | 20.00% | 20.00% | 30.00% |
| 10% | Test10_10%_ | 20.00% | 30.00% | 30.00% |
|  | Test20_10%_ | 20.00% | 20.00% | 30.00% |
| 20% | Test10_20%_ | 20.00% | 20.00% | 20.00% |
|  | Test20_20%_ | 20.00% | 20.00% | 30.00% |
| 40% | Test10_40%_ | 20.00% | 20.00% | 40.00% |
|  | Test20_40%_ | 25.00% | 25.00% | 40.00% |
| 60% | Test10_60%_ | 20.00% | 20.00% | 20.00% |
|  | Test20_60%_ | 15.00% | 20.00% | 30.00% |
| 80% | Test10_80%_ | 20.00% | 20.00% | 30.00% |
|  | Test20_80%_ | 25.00% | 30.00% | 35.00% |
| 100% | Test10_100%_ | 20.00% | 30.00% | 40.00% |
|  | Test20_100%_ | 30.00% | 35.00% | 40.00% |
